# Vine Cane Compounds to Prevent Skin Cells Aging through Solid Lipid Nanoparticles

**DOI:** 10.3390/pharmaceutics14020240

**Published:** 2022-01-20

**Authors:** Adriana Pereira, Maria João Ramalho, Renata Silva, Vera Silva, Rita Marques-Oliveira, Ana Catarina Silva, Maria Carmo Pereira, Joana A. Loureiro

**Affiliations:** 1 LEPABE (Laboratory for Process Engineering, Environment, Biotechnology and Energy), Department of Chemical Engineering, Faculty of Engineering, University of Porto, 4200-465 Porto, Portugal; up201505827@edu.fe.up.pt (A.P.); mjramalho@fe.up.pt (M.J.R.); 2 Associate Laboratory i4HB—Institute for Health and Bioeconomy, Faculty of Pharmacy, University of Porto, 4050-313 Porto, Portugal; rsilva@ff.up.pt (R.S.); veralssilva17@gmail.com (V.S.); ritoliveira.m@gmail.com (R.M.-O.); 3 UCIBIO (Applied Molecular Biosciences Unit), REQUIMTE, Laboratory of Toxicology, Department of Biological Sciences, Faculty of Pharmacy, University of Porto, 4050-313 Porto, Portugal; 4 UCIBIO, REQUIMTE, MEDTECH, Laboratory of Pharmaceutical Technology, Department of Drug Sciences, Faculty of Pharmacy, University of Porto, 4050-313 Porto, Portugal; 5 FP-ENAS (UFP Energy, Environment and Health Research Unit), CEBIMED (Biomedical Research Centre), Faculty of Health Sciences, University Fernando Pessoa, 4249-004 Porto, Portugal

**Keywords:** free radicals, natural compounds, lipid nanoparticles, skin aging, antioxidant activity, circular economy

## Abstract

The long lifespan of the world’s population has been raising interest in the research for new solutions to delay the aging process. With the aim of skin aging prevention, solid lipid nanoparticles (SLNs) were developed in this work for the encapsulation of three lipophilic natural compounds extracted from vine cane—epigallocatechin gallate (EGCG), resveratrol and myricetin. The developed loaded-SLNs proved to be stable, maintaining their adequate physicochemical characteristics for 30 days. In addition, the loaded-SLNs formulations exhibited high encapsulation efficiencies and loading capacities and high intracellular antioxidant activity. The mixture of EGCG-loaded SLNs with resveratrol-loaded SLNs proved to have the highest protection against induced oxidative stress. The in vitro cytotoxicity of the loaded SLNs was also evaluated, showing that the developed formulations are biocompatible for concentrations up to 50 µg/mL and could be safe for use in cosmetics. The encapsulation of EGCG, resveratrol and myricetin in SLNs seems to be a suitable strategy for the delivery of these antioxidants to the skin, improving their bioavailability.

## 1. Introduction

The world’s population is becoming older, and the consequences of aging are gaining special attention. It is believed that in 2050 the world’s population over 60 years will almost double, from 12% to 22% [1]. Skin aging occurs due to intrinsic and extrinsic factors. The first process is inevitable, varies from individual to individual and is genetically determined. Intrinsic aging is primarily caused by oxidative damage induced by free radicals or reactive oxygen species (ROS) [2]. ROS react with DNA components, leading to DNA mutations that can compromise the cell viability. These mutations can also induce protein aggregation and lipid peroxidation, among other harmful processes that lead to cell damage, homeostatic disruption and, finally, premature cell aging and cell apoptosis [3]. Although ROS play a key role in normal cell signal transduction and cell cycling, high ROS levels can cause tissue and organ damage, being associated with the progression of several health conditions and diseases, such as inflammatory diseases [4], cancer [5], and stroke [6]**,** among others.

As for the extrinsic aging process, it is caused by factors that can be controlled by humans, such as smoking, excessive consumption of alcoholic beverages, poor nutrition, and chronic sun exposure. This last factor is the main one responsible for skin deterioration over time, with 80% of facial skin aging resulting from prolonged sun exposure [7,8]. As with intrinsic aging, the direct production of ROS or free radicals due to sun exposure or other factors contributes to the aging process. These free radicals have the power to degrade collagen, increase the thickness of elastin and its folding, and induce proteins that degrade the cell matrix [9].

With the aging process, ROS production levels in skin cells increase and the ability to repair damaged DNA decreases. Therefore, the use of antioxidants to control ROS levels can prevent premature skin aging. In recent years, natural compounds are being explored by the cosmetic industry, due to their ability to scavenge free radicals and, therefore, to control the cellular redox imbalance [10].

Natural compounds with antioxidant activity can be extracted from different sources [11], such as grapes. Usually, after grape harvesting, the vine canes are burned or used as natural fertilizers. To make vine cane more profitable, new applications are currently being studied, such as its use as a source of bioactive compounds [12]. Three of the main compounds present in the vine cane are epigallocatechin gallate (EGCG), resveratrol and myricetin. EGCG is a polyphenol present in green tea (*Camelia sinensis*) and grapes, acting as a secondary metabolite; resveratrol is a polyphenolic flavonoid that is present in more than 70 species of plants, in blackberries, peanuts, grapes and rhubarb; and myricetin is a flavonoid present in many foods and drinks such as onions, grapes, berries, and red wine, among others [13,14,15]. These three natural compounds are known not only for their antioxidant activity but also for their anti-carcinogenic, anti-inflammatory and anti-obesity properties [14,16,17]. Despite these health-promoting properties, their low bioavailability and limited stability lead to their poor cellular absorption, limiting their use in cosmetics and food supplements [16,17,18].

The skin provides the largest body interface for administering drugs and for applying cosmetics, although it also acts as a barrier. The outermost skin barrier is the *stratum corneum* (SC) and constitutes a physical and chemical obstacle for external compounds [19]. The passage of active compounds through this lipid barrier depends on the molecules’ properties, physicochemical characteristics, such as size, surface charge, and lipophilicity, but also depends on the skin type and health conditions [20]. In the last years, nanotechnology provided an important contribution to enhancing the permeability of different types of compounds throughout the skin layers. Nanocarriers have the ability to increase molecular stability and protect the compound against aggressive agents, enable a controlled release, increase bioavailability and are considered non-toxic and biocompatible [21]. For the formulation to be able to penetrate the lipid layer, it should have a lipophilic composition [22]. Since lipid nanoparticles are capable of carrying lipophilic molecules, the encapsulation of natural compounds in these nanocarriers provides them with the ability to cross the SC. The lipid nanoparticles’ physiological composition interacts with SC making it rearrange the lipid molecules, letting the natural compounds penetrate the internal layers of the skin [22]. Additionally, the application of these types of nanoparticles promotes skin hydration due to the formation of an occlusive film preventing water loss from the SC and a reinforcement of the SC lipid structure [22]. For cosmetic applications, lipid nanoparticles should present dimensions between 200 and 300 nm to avoid systemic absorption [23]. Solid lipid nanoparticles (SLNs) are composed of lipids that are solid at both room and body temperature, where the encapsulated compound is usually dissolved, surrounded by a layer of emulsifier(s) that stabilizes the nanoparticles, decreasing the surface tension [24].

In this work, SLNs formulations were developed for the delivery of the main components of the vine cane—EGCG, myricetin and resveratrol. The final aim was to help to control the cellular imbalance of ROS, preventing premature skin aging, and by using the subproducts of vine cane, it will be possible to contribute to the circular economy.

## 2. Materials and Methods

### 2.1. Materials

Epigallocatechin Gallate (EGCG, ()-epigallocatechin-3-gallate, ≥94% purity, molecular weight 458 g/mol) was purchased from Kosher, myricetin (3,3′,4′,5,5′,7-Hexahydroxyflavone, ≥97% purity, molecular weight 318,24 g/mol) and resveratrol (trans-1,2-(3,4′,5-Trihydroxydiphenyl)ethylene, ≥99% purity, molecular weight 228,25 g/mol) were both purchased from TCI Chemicals, Tokyo, Japan. Precirol^®^ 5 ATO (glyceryl distearate), Gelucire^®^ 39/01, Cetyl palmitate, Suppocire DM Pellets, Compritol^®^ HD5 ATO, Gelucire^®^ 50/13 (Stearoyl polyoxyl-32 glycerides), Suppocire NA15 Pellets, Gelucire^®^ 43/01 (hard fat compounds) and Apifil^®^ (PEG-8 beeswax) were acquired from Gattefossé (Nanterre, France), Dynasan^®^ 114 (glyceryl tristearate), Softisan^®^ 100 (hydrogenated coco-glycerides) and Softisan^®^ 154 (hydrogenated palm oil) were purchased from IOI Oleo GmbH (Hamburg, Germany), Witepsol^®^ E76 (hard fat compounds) was acquired from Oxi-Med (Barcelona, Spain) and Glyceryl monostearate and Stearic acid from Acopharma S.A. (Terrassa, Spain). Pluronic^®^ F-127 (MW 12,600 g/mol), pure anhydrous ethanol, (MW 46,07 g/mol), HEPES hemisodium salt (pH 7,4, MW 249,30 g/mol), 2,2-diphenyl-1-picrylhydrazyl (DPPH, MW 394,32 g/mol) and uranyl acetate (≥98 % purity, MW 424,15 g/mol) were purchased from Sigma-Aldrich. St. Louis, MO, USA For the in vitro cytotoxicity assay, Dulbecco’s modified Eagle’s medium (DMEM) with 4.5 g/L glucose and GlutaMAX™, fetal bovine serum (FBS), 0.25% trypsin/1 mM EDTA and Hanks’ balanced salt solution (HBSS) without calcium and magnesium [HBSS (−/−)] were obtained from Gibco^TM^ (Thermo Fisher Scientific, Cleveland, OH, USA). Neutral red (NR) solution, tert-butyl hydroperoxide (t-BHP) solution, 2′,7′-Dichlorofluorescin diacetate (DCFH-DA) and Triton™ X-100 detergent solution were obtained from Sigma-Aldrich. Antibiotic (10,000 U/mL penicillin, 10,000 μg/mL streptomycin) was obtained from Biochrom (Cambridge, UK). Dimethyl sulfoxide (DMSO) was obtained from Merck (Darmstadt, Germany). All sterile plastic material was obtained from Corning Costar (Cambridge, MA, USA).

### 2.2. Methods

#### 2.2.1. Formulation Studies

##### Lipid Screening

For the SLN production it is important to select a solid lipid that has the capacity to solubilize the total amount of active compound. The adequate lipid screening indicates the optimal ratio between the solid lipid and the active compound. Therefore, each solid lipid was tested to assess its ability to dissolve the three active compounds of interest. The experimental procedure was based on a process described by Tichota et al. [25]. Briefly, 100 mg of each solid lipid was mixed with 1 mg of the natural compounds. If the lipid did not dissolve the compound in this proportion, a 1:200 ratio was tested. The mixture of lipid/natural compound was heated up to 80 °C (temperature above the solid lipid melting point) for 1 h with magnetic agitation. The solubility of the natural compounds was visually determined, at 15-min intervals, observing the presence or absence of crystals of the compounds in the lipid. Additionally, the presence of aggregates was determined by dynamic light scattering (DLS) using a Malvern Zetasizer Nano ZS (Malvern Instrument, Malvern, UK) at 80 °C during the required heating period.

To evaluate the NCs stability above the melting point temperature, the UV-Vis absorbance at the characteristic wavelength of each compound (274 nm for EGCG, 305 nm for resveratrol and 377 nm for myricetin) (BioTek Synergy HT Microplate Reader, BioTek, Winooski, VT, USA) was measured after 10 min of heating at 80 °C.

##### Emulsifier Compatibility Evaluation

To select the most suitable emulsifier, unloaded SLNs without the natural compounds were prepared according to the experimental procedure detailed in the following section (2.2.2). Two different emulsifiers, commonly used in formulations of lipid nanoparticles, were tested: pluronic-F127 (non-ionic copolymer) and tween 80 (also known as polysorbate 80—non-ionic) [24,26]. Furthermore, different Ultra-Turrax T25 (0.5 s; 1 and 2 min) and sonication (5, 10, 15 and 30 min) times were tested to select the best experimental conditions. The produced nanoparticles were physiochemical characterized to evaluate if they had the required characteristics to be chosen for final formulation (more details in Section 2.2.3). The physicochemical properties of the produced SLNs were monitored over 138 days to evaluate their stability at storage conditions.

#### 2.2.2. Nanoparticle Preparation

Three different formulations were prepared, (i) EGCG loaded NLCs, (ii) resveratrol loaded NLCs, and (iii) myricetin loaded NLCs. Nanoparticles were produced by the high shear homogenization followed by ultrasonication. The experimental preparation was based on a procedure described by Vaz et al. [27]. First, a mixture of 1 g of the solid lipid and 20 mg of the selected natural compound was heated at 80 °C (5 to 10 °C above the melting point of the lipid) until complete melting. A total of 8.7 mL of an aqueous solution (10% *w*/*v*) of the emulsifier selected was heated separately. When the lipid solution was completely melted and both solutions were at the same temperature, the aqueous phase was added to the lipidic phase.

For homogenization, the resultant mixture was stirred in the Ultra-Turrax T25 (Janke and Kunkel IKA-Labortechnik, Staufen, Germany) at 13,500 rpm, followed by sonication at an amplitude of 80% and an ultrasonic frequency of 24 kHz (Vibra-Cell^TM^ CV15, Sonics and Materials, Newtown, CT, USA). The samples were cooled down to room temperature, with magnetic agitation (100 rpm), and stored protected from the light. SLNs were produced in triplicate and un-loaded SLNs were also prepared as control samples.

#### 2.2.3. Nanoparticle Characterization

Nanoparticles were characterized in terms of size, zeta potential (ZP) and morphology. The mean particle size, polydispersity index (PdI) and ZP were measured by DLS using a Malvern Zetasizer Nano ZS (Malvern Instrument, Malvern, UK) on days 1, 7, 14 and 30, to evaluate their stability [28]. Before measurements, the formulations were diluted in ultrapure water (1:100 *v*/*v*) and every measurement was run in triplicate. For size determination, the samples were placed in a polystyrene cuvette (DTS0012, Malvern Instrument, UK) and analyzed with a 633 nm red laser at 25 °C with a backscattering angle of 173°. The refractive index (1.330) and viscosity (0.8872 cP) of water as the dispersant were used. Lipid refractive index and absorption were set up at 1.4 and 0.00, respectively. The obtained results were given as intensity distribution. ZP measurements were performed using a folded capillary cell (DTS1070, Malvern Instrument, UK) and the values were obtained by the Smoluchowski model using the dielectric constant (78.5), refractive index (1.330), and viscosity (0.8872 cP) of water as the dispersant at 25 °C. The morphological analysis was performed by transmission electron microscopy (TEM) using a JEM-1400 electron microscope (JEOL, Tokyo, Japan) with an accelerating voltage of 80 kV. The experimental procedure was based on a procedure described [29]. Briefly, 5 µL of each sample were deposited on a 400-mesh carbon-formvar copper grid (Agar Scientific, London, Essex, UK) and left to absorb for 5 min. Then, the samples were stained with 10 µL of 2% (*w*/*v*) uranyl acetate solution and left to air-dry before analysis.

#### 2.2.4. Determination of Encapsulation Efficiency and Loading Capacity

The encapsulation efficiency (EE) and the loading capacity (LC) of the three tested natural compounds in the SLNs were determined indirectly. To separate the free compounds from the loaded SLNs, the samples were diluted in ultrapure water (1:200) and subsequently filtered (Amicon Ultra Centrifugal Filters Ultracell-30 kD, Merck Millipore Ltd., Burlington, MA, USA Tullagreen, Carrigtwohill; from Sigma-Aldrich) at 14,000× *g* for 3 min. After the centrifugation, the filtrate containing the free compounds was collected and mixed with ethanol 100% (*v*/*v*) at a volume ratio of 3:7. The amount of free natural compound was individually quantified by UV-Vis absorbance at the characteristic wavelength of each compound (274 nm for EGCG, 305 nm for resveratrol and 377 nm for myricetin) (BioTek Synergy HT Microplate Reader, BioTek, UK). Previously, the maximum wavelength of each compound was determined, and a calibration curve was drawn for each compound. The concentration of free compounds was determined from a calibration curve in ethanol 100% (*v*/*v*) at a volume ratio of 3:7 (water). This experiment was run in triplicate for each type of loaded-SLNs and the EE was determined as described in the following equation (Equation (1)).

EE (%) = (Total amount of compound in formulation-amount of free compound)/(Total amount of compound in formulation) x 100(1)

The LC was determined following the equation below (Equation (2)).

LC (%) = (Total mass of natural compound encapsulated)/(Total mass of SLN+ Total mass of natural compound encapsulated) x 100(2)

#### 2.2.5. Antioxidant Assay

The antioxidant activity of the natural compounds was evaluated using the DPPH methodology. Additionally, to the three natural compounds alone, mixtures of two or three compounds were also tested to assess possible synergies. DPPH is a stable free radical with a deep violet color and has a strong absorption band at 517 nm. When in contact with an antioxidant substance, DPPH’s color vanishes to a yellow pale as the electron pairs off [30].

The experimental procedure was based on a procedure previously described by Duan et al. (2006) [31]. Firstly, stock solutions were prepared by dissolving the natural compounds in methanol. Each stock solution was added to 175 µL of the DPPH methanolic solution (0.16 mM) to obtain a final concentration of natural compound or mixture of 4 µM and a final volume of 200 µL. The studied mixtures were: EGCG + myricetin; EGCG + resveratrol, resveratrol + myricetin; and resveratrol + myricetin + EGCG. The solutions were vortexed for 1 min and then were kept in the dark for 30 min. Finally, the UV-Vis absorbance was measured at 517 nm (BioTek Synergy HT Microplate Reader, BioTek, UK) and the scavenging potential was determined with the following equation (Equation (3)).

Scavenging effect (%) = (1 − (A_sample_ − A_sample blank_)/A_control_) × 100 (3)

where A_control_ is the absorbance of the control (DPPH solution without the sample), A_sample blank_ is the absorbance of the sample without DPPH and A_sample_ is the absorbance of the test sample (DPPH with the mixtures or natural compound).

#### 2.2.6. Accelerated Stability

To study possible changes that may occur during storage, an accelerated stability assay was performed. The procedure was adapted from a previous described protocol [32]: the loaded SLNs were diluted in ultrapure water (1:100 *v*/*v*) and submitted to two centrifugation steps (MiniSpin Eppendorf centrifuge) at 3000× *g* for 30 min at room temperature. After each cycle, the samples were visually examined to verify the occurrence of phase separation, creaming or flocculation, and analyzed in terms of particle size, PdI and ZP (more details in Section 2.2.3).

#### 2.2.7. In Vitro Cytotoxicity Assay and Antioxidant Assay

##### Cell Culture

Immortalized human keratinocytes (HaCaT cells), an in vitro model widely used for biological and medical researches [33], were used in this work. These cells were cultured in 75 cm^2^ flasks using DMEM with 4.5 g/L glucose and GlutaMAX™, supplemented with 10% FBS, 100 U/mL penicillin and 100 μg/mL streptomycin. The cells were kept in a 5% CO_2_ 95% air atmosphere, at 37 °C, and the cell culture medium was changed every 2 days. When at 80–90% of confluence, cultures were passaged by trypsinization (0.25% trypsin/1 mM EDTA). For the experiments, the cells were always seeded in 96-well plates at a density of 20,000 cells/well. The cells used in all experiments were taken between the 42nd and 50th passages.

##### Cytotoxicity of the Formulations

The assay was based on a procedure previously described by Vaz et al. (2019) [27]. Briefly, 24 h after seeding, the cells were exposed to the formulations (0–1000 µg/mL) prepared in fresh cell culture medium. Twenty-four hours after treatment, cytotoxicity was evaluated by the neutral red (NR) uptake assay. Triton™ X-100 (1%) was used as a positive control. The cell culture medium was aspirated, followed by the addition of fresh cell culture medium containing NR (50 μg/mL). The cells were then incubated, at 37 °C, in a humidified 5% CO_2_—95% air atmosphere, for 90 min. After incubation, the cell culture medium was removed, and the NR dye retained only by viable cells was extracted with lysis buffer (absolute ethyl alcohol/distilled water (1:1) with 5% acetic acid). The absorbance was measured, at 540 nm, in a multiwell plate reader (PowerWaveX BioTek Instruments, Vermont, USA). The percentage of NR uptake relatively to that of the control cells (0% formulation present) was used as the cytotoxicity measure. Four independent experiments were performed in triplicate. The cytotoxicity of the free drugs (0.81 µg/mL) was also evaluated by the NR uptake assay, 24 h after exposure, as performed for the evaluation of the formulation’s cytotoxicity.

##### Evaluation of the Protection of the Formulations against Oxidative Stress: Effects on t-BHP Induced Increase in ROS Levels

The antioxidant effect of the developed formulations was evaluated in terms of their ability to protect against t-BHP-induced increase in the intracellular levels of ROS and reactive nitrogen species (RNS). For that purpose, the DCFH-DA probe was used, which is hydrolyzed once in the cytoplasm, leading to the formation of 2′,7′-dichlorodihydrofluorescein (DCFH). In the presence of ROS/RNS, DCFH is oxidized to the highly fluorescent 2′,7′-dichlorofluorescein (DCF), which can be quantified and which fluorescence intensity is proportional to the levels of ROS/RNS [34]. Briefly, HaCaT cells were seeded in 96-well plates (20,000 cells/well) and, 24 h after seeding, the cells were pre-incubated with 20 µM DCFH-DA, protected from light, at 37 °C, in a 5% CO_2_—95% air atmosphere. After 60 min of pre-incubation, DCFH-DA was removed and the cells exposed to tert-butyl hydroperoxide (0–500 µM) in the presence or absence of the tested formulations (alone or in mixtures, 50 µg/mL), at 37 °C, in a 5% CO_2_—95% air atmosphere. After 24 h of exposure, the fluorescence was measured at 485 nm excitation and 530 nm emission wavelengths in a multiwell plate reader (PowerWaveX BioTek Instruments, VT, USA). Four independent experiments were performed in triplicate.

#### 2.2.8. Statistical Analysis

All statistical calculations were performed using the GraphPad Prism 8 for Windows (GraphPad Software, San Diego, CA, USA). The normality of the data distribution was assessed using the KS, D’Agostino and Pearson omnibus, and Shapiro–Wilk normality tests. For data with a parametric distribution, one-way ANOVA was used to perform the statistical comparisons, followed by Dunnett’s multiple comparisons test. For data with a non-parametric distribution, the Kruskal–Wallis test was used to perform the statistical comparisons, followed by Dunn’s multiple comparisons test. In the evaluation of the antioxidant effect of the formulations, the comparisons were performed using two-way ANOVA followed by Tukey’s multiple comparisons test. Details of the performed statistical analysis are described in the figure legend. Differences were significant for *p*-values lower than 0.05.

## 3. Results and Discussion

### 3.1. Formulation Studies

To assess the compatibility between the lipids and the natural compounds, a lipid screening was made. These lipids were selected due to their biocompatibility with the skin since the aim of this work was to develop a formulation for cosmetic application for skin aging prevention. From the 15 solid lipids tested, only 2 dissolved the 3 natural compounds: Gelucire^®^ 50/13 and Compritol^®^ HD5 ATO (Supplementary Table S1 and Supplementary Figure S1 and Supplementary Figure S2).

To select the best emulsifier (Tween^®^80 or Pluronic-F127) and to optimize the production process, SLNs were first produced without the natural compounds. Different combinations of Ultra-Turrax (UT) (0.5 and 2 min) and sonication (S) (5, 15 and 30 min) times were tested, and the samples were visually analyzed and characterized by DLS. The combination of Gelucire^®^ 50/13 with the emulsifiers Pluronic-F127 or Tween^®^ 80 presented an inadequate consistency (Supplementary Table S2 and Supplementary Table S3)) and were, therefore, not suitable for the encapsulation of the natural compounds. The SLN formulation produced with Compritol^®^ HD5 ATO and Tween^®^ 80 were polydisperse, thus not suitable for skin application (Supplementary Table S5).

**Figure 1:**
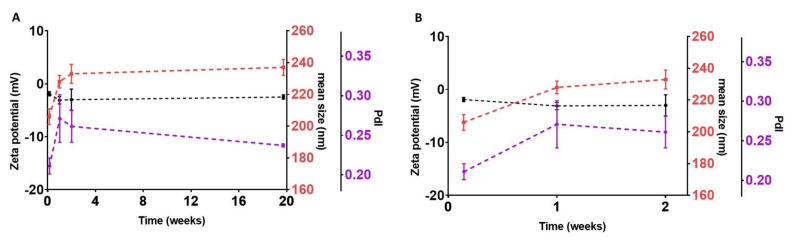
(**A**) Stability of the selected unloaded SLNs formulation at days 1, 7, 14 and 138. An enlargement of the first 14 days is presented in (**B**). Black line: Graphical representation of variations in ZP values (data plotted on the left Y-axis); Red line: Graphical representation of variations in mean sizes (data plotted on the first right Y-axis); Purple line: Graphical representation of variations in PdI values (data plotted on the second right Y-axis). Results are given as mean ± SD (*n* = 3).

After testing the possible combinations varying the Ultra-Turrax and ultrasonication times, one combination composed of Compritol^®^ HD5 ATO and Pluronic-F127 exhibited the desired characteristics after 2 min in the Ultra-Turrax and 15 min in the ultrasonicator (Supplementary data, Table S4). The prepared SLNs were analyzed by DLS, and one day after production, their size and ZP were 206 ± 5 nm and −1.9 ± 0.4 mV, respectively. Furthermore, the PdI was below 0.3, which was indicative that the formulation was monodispersed. Therefore, this combination was suitable for the final aim of the formulation. The chosen combination was studied to assess its stability over time. The size, PdI and ZP of the SLNs were studied on days 1, 7, 14 and 138. In Figure 1, the results of the stability studies are presented. No degradation of any of the natural compounds was observed after 10 min at the temperature above the Compritol^®^ HD5 ATO melting point.

As the results show, the size increased 22 nm between day 1 and day 7 (*p* < 0.05), probably because the nanoparticles were not fully stabilized in the day after their production. Nonetheless, after day 7, the size remained constant (*p* > 0.05). The PdI was below 0.3 (meaning a monodispersed solution), and the ZP was stable over time (*p* > 0.05).

### 3.2. Nanoparticle Characterization and Stability Studies

**Figure 2:**
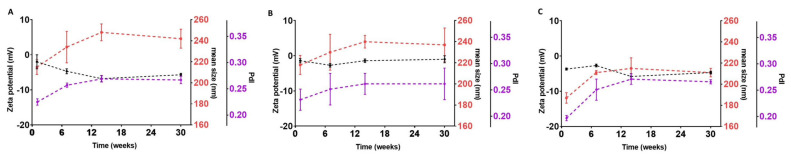
Stability of the SLNs (composed by the solid lipid Compritol^®^ HD5 ATO 345 and the emulsifier Pluronic-F127) loaded with (**A**) EGCG, (**B**) resveratrol or (**C**) myricetin. Black line: Graphical representation of variations in ZP values (data plotted on the left Y-axis); Red line: Graphical representation of variations in mean sizes (data plotted on the first right Y-axis); Purple line: Graphical representation of variations in PdI values (data plotted on the second right Y-axis). Results are given as mean ± SD (*n* = 3).

After selecting the most suitable formulation parameters, different SLNs loaded with the natural compounds—EGCG, resveratrol and myricetin—were prepared. The stability of the developed SLNs was evaluated for 30 days. The size, ZP and PdI of the loaded-SLNs were analyzed. In Figure 2, the mean and standard deviation (SD) of the size, ZP and PdI for each type of loaded SLN (EGCG, resveratrol and myricetin) is presented.

Similar to that observed for unloaded SLNs, the size of EGCG-loaded, resveratrol-loaded and myricetin-loaded SLNs slightly increased between day 1 and day 7. After day 7, the size remained constant until the end of the stability study (*p* > 0.05). Furthermore, it is possible to conclude that, at the end of the study, all SLNs formulations maintained the ideal size and proved to be stable over time, with no signs of aggregation observed.

All prepared loaded-SLNs presented a PdI below 0.3, which is the threshold value for the cosmetic use of this type of nanoparticle [35]. Thus, it is possible to conclude that all samples are monodisperse, meaning that there were no significant size variations. Furthermore, there were no significant changes in the PdI values over time.

In terms of ZP, similar to unloaded SLNs, all samples exhibited slightly negative values (−2.0 ± 2.0 mV for EGCG-SLNs; −3.7 ± 0.3 mV for myricetin-SLNs; for –1.4 ± 0.6 mV for resveratrol-SLNs). The obtained ZP values suggest that the natural compounds are entrapped in the lipid core of the SLNs, instead of being adsorbed to the SLNs’ surface. This slightly negative surface charge is advantageous regarding NPs’ biocompatibility. Positively charged NPs are usually associated with higher toxicity. Furthermore, as these nanoparticles have a lipid core, it is expected that they interact with the skin, releasing their content. Indeed, several authors reported the ability of negatively charged lipid nanoparticles to permeate human/animal skin [36,37,38].

Despite ZP values being close to zero, no nanoparticle aggregation was observed since the emulsifier Pluronic-F127 provided steric and electrostatic stabilization [39]. The emulsifier layer on the SLNs surface exerts a masking effect of the surface charges, which can also justify the attained low ZP values. Overall, the ZP of each type of SLN did not change significantly (*p* > 0.05).

### 3.3. Transmission Electron Microscopy (TEM)

**Figure 3:**
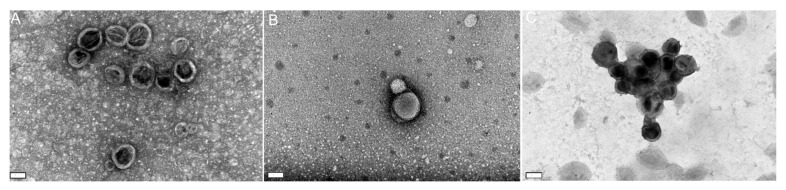
TEM images of the (**A**) EGCG-loaded, (**B**) resveratrol-loaded and (**C**) myricetin-loaded SLNs. The scale bar corresponds to 100 nm.

In Figure 3, images of the loaded SLNs obtained by TEM are presented.

From Figure 3A–C, it is possible to observe that all prepared SLNs have a typical spherical shape. Furthermore, the nanoparticles have a size slightly smaller than the values obtained in the DLS, which may be related to the nanoparticle impregnation technique necessary for TEM analysis. Other authors also reported mean sizes measured by DLS slightly higher than the ones obtained by TEM, due to the interference of the dispersant in the hydrodynamic diameter [40].

### 3.4. Encapsulation Efficiency and Loading Capacity

Table 1 presents the mean values of the EE and LC values for each of the produced loaded SLNs.

The EE in the SLNs was high for all natural compounds. These values were expected since both resveratrol and myricetin are lipophilic compounds and, therefore, have an affinity to the lipid matrix [19,20,24].

Regarding LC, the solubility of the natural compounds in the selected lipids is essential to obtain high values [41,42]. The proper selection of the lipids and emulsifiers through the lipid screening and emulsifier optimization processes positively affected the LC; hence, the obtained values are close to the theoretical LC (1.058). These EE and DL results demonstrate that the developed SLNs can be effective nanocarriers for the encapsulation and delivery of natural compounds with low bioavailability. In addition, these high values demonstrate that the lipid screening, the emulsifier optimization and the production of the SLNs were successful. In addition, despite the low LC, our in vitro antioxidant study in human keratinocytes (please see Section 3.7 showed that all loaded SLN formulations provide protection against induced oxidative stress by significantly decreasing the ROS/RNS levels.

### 3.5. Antioxidant Assay

An in vitro antioxidant assay was performed to assess the antioxidant activity of each compound and mixtures between the three compounds. In Table 2, the mean antioxidant activity percentages obtained for the natural compounds are presented.

All three natural compounds presented an antioxidant activity at the studied concentration (4 µM). The results showed that EGCG presented the highest antioxidant activity and resveratrol the lowest one (*p* < 0.05).

The EGCG:resveratrol mixture and the mixture of the three compounds presented a synergic effect, where the experimental value was higher than the average value of the mixtures indicating two promising interactions (*p* < 0.05). As they are the ones displaying higher synergistic effect, these two combinations were chosen to evaluate the in vitro antioxidant activity in human skin cells. The other two mixtures (EGCG: myricetin and resveratrol:myricetin) presented an approximated null interaction since the experimental value obtained was very close to the theoretical one (*p* > 0.05).

### 3.6. Accelerated Stability

**Figure 4:**
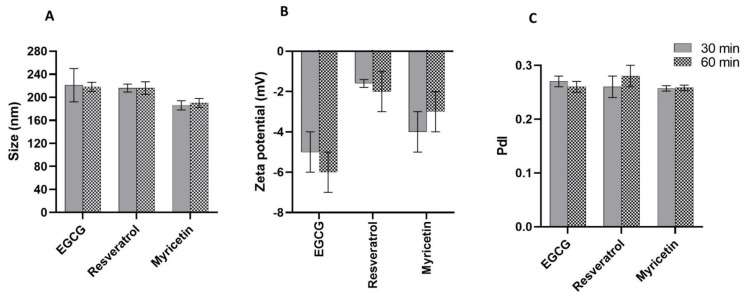
Mean particle size (**A**), zeta potential (ZP) (**B**) and polydispersity index (PdI) (**C**), of the EGCG, resveratrol and myricetin-loaded SLNs after the accelerated stability assay (mean ± SD, *n* = 3).

Accelerated stability by centrifugation was performed to estimate changes that may occur during storage of the formulations at room temperature, anticipating stability problems over time, including phase separation, creaming, flocculation or other signs of instability. Mechanical stress applied during centrifugation is often used to estimate the physical stability of heterogeneous systems, such as colloidal dispersions of lipid nanoparticles. In Figure 4, the mean particle size, PdI and ZP values for each sample after the two centrifugations are displayed.

From Figure 4, it can be seen that no significant changes were observed in the sizes, PdI and ZP values, between the two centrifugations, for all the studied SLNs. These results demonstrate that both centrifugations did not significantly affect the stability of the formulations. Visually, the formulations appeared to be stable and homogeneous after each centrifugation (Supplementary Figure S3). No signs of creaming, flocculation or phase separation were detected, indicating good long-term stability of the tested formulations, suggesting that these SLNs are adequate for incorporation in cosmetic formulations.

### 3.7. In Vitro Cytotoxicity Assay and Antioxidant Evaluation

#### 3.7.1. Cytotoxicity of the Formulations

**Figure 5:**
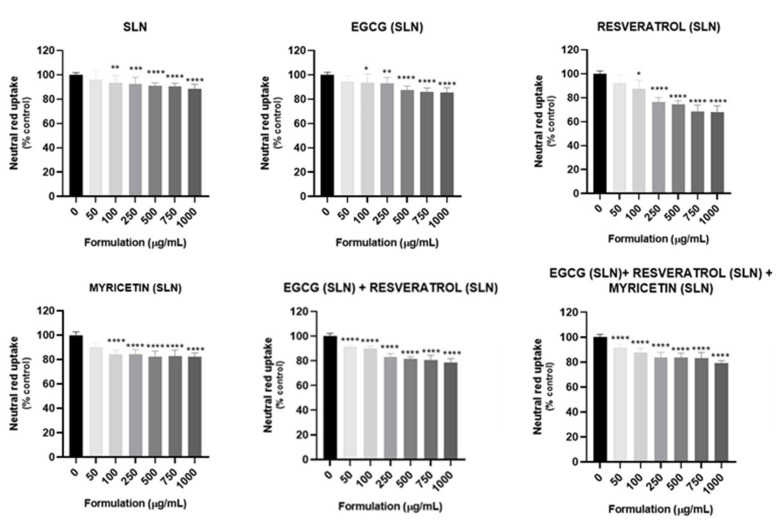
Cytotoxicity of the developed formulations evaluated in HaCat cells by the neutral red (NR) uptake assay, 24 h after exposure. Results are expressed as Mean ± SD from 4 independent experiences, performed in triplicate. Statistical comparisons were made using one-way ANOVA followed by Tukey’s multiple comparisons test (for data with a parametric distribution) or using the Kruskal–Wallis test followed by Dunn’s multiple comparisons test (for data with a non-parametric distribution) (* *p* < 0.05; ** *p* < 0.01; *** *p* < 0.001; **** *p* < 0.0001 for each formulation vs. 0 μg/mL). In all cases, *p*-values < 0.05 were considered significant.

In vitro cytotoxicity assays were performed to select the non-cytotoxic concentrations to be used in the evaluation of the antioxidant effects of the formulations against t-BHP-induced oxidative stress. The obtained results for the NR uptake assay are presented in Figure 5.

A concentration-dependent reduction in NR uptake was observed for all the formulations, including the unloaded formulation. In accordance with the obtained data, no significant cytotoxicity was detected after 24 h exposure to unloaded, or SLNs loaded with EGCG, resveratrol or myricetin at the SLN concentration of 50 µg/mL. However, a significant reduction in NR uptake was observed for concentrations equal or higher than 100 µg/mL. For the EGCG:resveratrol and the EGCG:resveratrol:myricetin SLNs formulations, a significant reduction in the NR uptake was detected for all the tested concentrations. However, although significant, the NR uptake observed 24 h after exposure to the EGCG:resveratrol and EGCG:resveratrol:myricetin SLNs formulations at the 50 µg/mL concentration was always higher than 90%, when compared to control cells ((0 µg/mL) (NR uptake significantly decreased to 91.8% and 91.7% 24 h after exposure to 50 µg/mL EGCG:resveratrol and EGCG:resveratrol:myricetin SLNs formulations, respectively).

Based on the obtained results, the 50 µg/mL concentration was selected for the cell-based antioxidant assay since there was no remarkable cytotoxicity at this concentration.

**Figure 6:**
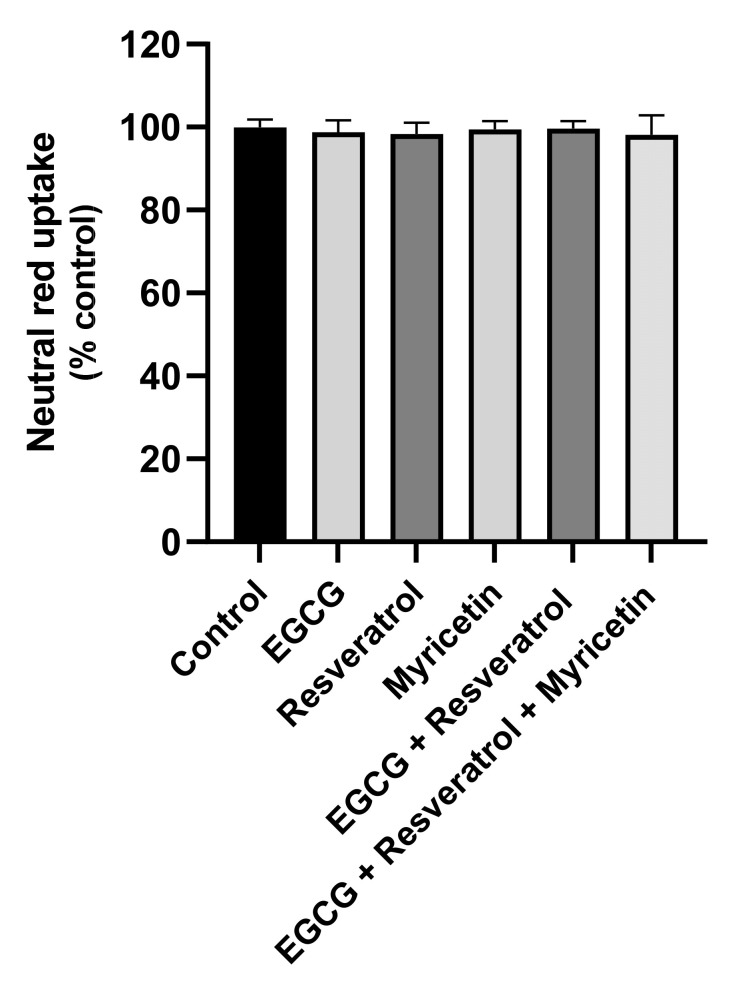
Cytotoxicity of the free drugs (alone or in combination) evaluated in HaCaT cells by the neutral red (NR) uptake assay 24 h after exposure. The compounds were only evaluated at the 0.81 µg/mL concentration, the concentration present in the 50 µg/mL concentration of each formulation. Results are expressed as mean ± SD from 4 independent experiences, performed in triplicate. Statistical comparisons were made using one-way ANOVA followed by Dunnett’s multiple comparisons test. In all cases, *p*-values < 0.05 were considered significant.

The cytotoxicity of the free compounds (alone or in the same mixtures presented in the developed formulations) was also evaluated but only at the 0.81 µg/mL concentration, the final concentration present in the 50 µg/mL concentration of each formulation. As observed in Figure 6, no significant effects on NR uptake were detected 24 h after exposure to all the tested active compounds, both alone and in combination, suggesting that this concentration is non-cytotoxic towards HaCaT cells.

#### 3.7.2. Evaluation of the Protection of the Formulations against Oxidative Stress: Effects on t-BHP Induced Increase in ROS Levels

**Figure 7:**
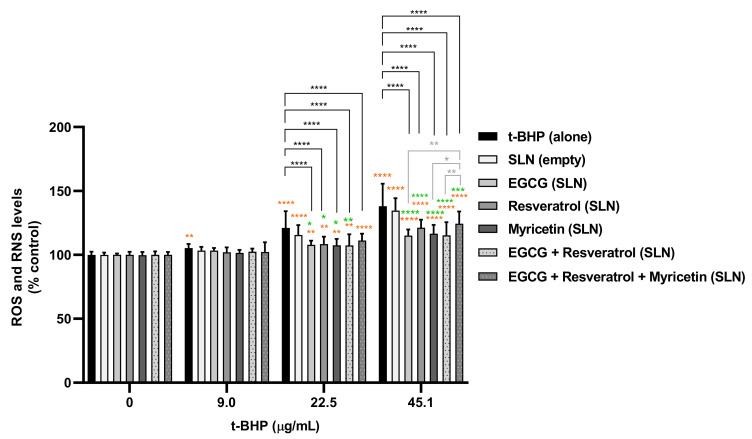
Intracellular levels of ROS/RNS evaluated in HaCaT cells 6 h after exposure to t-BHP (0–45.1 µg/mL) in the presence or absence of the developed SLN formulations. Results are expressed as mean ± SD from six independent experiments performed in triplicate. Statistical comparisons were made using two-way ANOVA followed by Tukey’s multiple comparisons test (* *p* < 0.05, ** *p* < 0.01, *** *p* < 0.001; **** *p* < 0.0001; orange represents the statistical analysis for each condition versus 0 µM t-BHP; green represents the statistical analysis at each t-BHP concentration for the comparison between NC-loaded SLN formulations and the unloaded SLN formulation; black represents the statistical analysis at each t-BHP concentration for the comparison between each SLN formulation and t-BHP alone; grey represents the statistical analysis at each t-BHP concentration for the comparison between the different NC-loaded SLN formulations). In all cases, *p*-values < 0.05 were considered significant.

**Figure 8:**
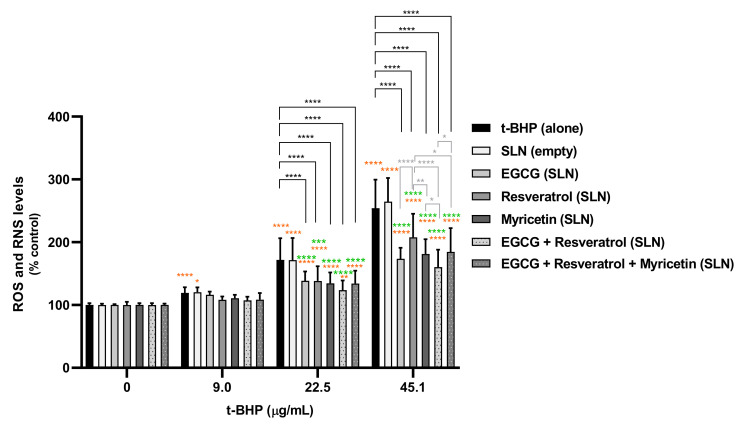
Intracellular levels of ROS/RNS evaluated in HaCaT cells 24 h after exposure to t-BHP (0–45.1 µg/mL) in the presence or absence of the developed SLN formulations. Results are expressed as mean ± SD from six independent experiments performed in triplicate. Statistical comparisons were made using two-way ANOVA followed by Tukey’s multiple comparisons test (* *p <* 0.05, ** *p <* 0.01, *** *p <* 0.001; **** *p <* 0.0001; orange represents the statistical analysis for each condition versus 0 µM t-BHP; green represents the statistical analysis at each t-BHP concentration for the comparison between NC-loaded SLN formulations and the unloaded SLN formulation; black represents the statistical analysis at each t-BHP concentration for the comparison between each SLN formulation and t-BHP alone; grey represents the statistical analysis at each t-BHP concentration for the comparison between the different NC-loaded SLN formulations). In all cases, *p*-values < 0.05 were considered significant.

The antioxidant protective effects of the developed SLNs formulations were further assessed in HaCat cells. The mixture of EGCG-loaded plus resveratrol-loaded SLNs and the mixture of all SLNs formulations (EGCG-loaded, resveratrol-loaded and myricetin-loaded) were also evaluated. HaCat cells were incubated with 0–45.1 µg/mL t-BHP (0–500 µM) in the presence or absence of the formulations (50 µg/mL) containing 0.81 µg/mL of the active compounds, and ROS/RNS intracellular levels were then quantified 6 and 24 h after exposure. The results are presented in Figure 7 and Figure 8.

As shown in Figure 8, t-BHP induced a significant and concentration-dependent increase in ROS/RNS intracellular levels for both 6 and 24 h after exposure. For 9.0 µg/mL t-BHP, it was verified that none of the loaded NLCs present an antioxidant effect against t-BHP-induced oxidative stress since a significant decrease in ROS/RNS levels was not observed. Furthermore, the simultaneous exposure to t-BHP and loaded SLN formulations at 22.5 µg/mL and 45.1 µg/mL resulted in a significant decrease in ROS/RNS production when compared to t-BHP alone, suggesting a protective antioxidant effect of these SLNs formulations against t-BHP-induced oxidative stress. That effect was more pronounced at 24 h after exposure, attesting that the formulation was more efficient after 24 h of contact with cells. From the treatment with only one compound-loaded SLNs formulation, the EGCG-loaded SLNs formulation was considered the most effective in decreasing ROS/RNS production. In addition, from all the SLNs-loaded formulations, the EGCG:resveratrol combination was responsible for the highest protection against t-BHP-induced oxidative stress. As expected, the unloaded SLNs formulation was not able to decrease the t-BHP-induced ROS/RNS production, suggesting that the observed protective effects observed for all the formulations seem to be a result of the antioxidant effects of the loaded natural compounds.

In addition, although not being the most efficient formulation, the mixture of the three loaded-SLNs formulations still proved to significantly improve the protective effects against oxidative stress. The obtained results suggest that the developed loaded SLNs are adequate for the development of novel cosmetic formulations with antioxidant activity.

## 4. Conclusions

SLNs for the delivery of photoprotective agents have attracted attention for cosmetics formulations due to their unique properties [43]. Envisaging a cosmetic application, different SLNs containing natural compounds—EGCG, myricetin and resveratrol—with antioxidant activity were successfully produced after optimization. The loaded-SLNs maintained adequate physicochemical properties for cosmetic applications over 30 days. Additionally, an accelerated stability assay suggested that these SLNs have higher storage stability. The EE obtained were high and the LC values were close to the theoretical value, revealing the ability of the SLNs to encapsulate the tested natural compounds, producing cost-effective and sustainable products.

Finally, in vitro cytotoxicity tests proved that the developed formulations are biocompatible with human skin cells. Of significance, all the developed formulations exhibited adequate antioxidant activity in human skin cells against t-BHP-induced oxidative stress for formulations containing 0.81 µg/mL of the natural molecules. The mixture of the loaded formulations was also evaluated, and the mixture of EGCG:resveratrol exhibited the highest protection against t-BHP-induced oxidative stress. To conclude, the developed SLNs demonstrated to be suitable nanocarriers for natural compounds, protecting them against external agents and improving bioavailability. Therefore, these loaded-SLNs could be used to develop new cosmetic products with health-promoting properties for skin aging prevention. Other authors presented SLNs, such as other lipid-based NPs, as adequate components for beauty products due to their ability to enhance the protective effects of antioxidant molecules [44,45,46]. Furthermore, this study highlights the application of the circular economy through the possibility of repurposing the vine cane waste into cosmetic formulations, generating more sustainable products.

## Figures and Tables

**Table 1:** Encapsulation efficiencies (EE) and loading capacity (LC) of EGCG, resveratrol and myricetin in the developed SLNs (mean ± SD, *n* = 3).

	EGCG	Resveratrol	Myricetin
EE (%)	90 ± 5	95.6 ± 0.9	98 ± 2
LC (%)	0.96 ± 0.05	1.01 ± 0.01	1.03 ± 0.02

**Table 2:** Antioxidant activities of EGCG, resveratrol (R), myricetin (M) and mixtures of EGCG:resveratrol, EGCG:myricetin, resveratrol:myricetin and EGCG:resveratrol:myricetin (mean ± SD, *n* = 3).

Scavenging Activity (%)	EGCG	R	M	EGCG:R	EGCG:M	R:M	EGCG:R:M
Obtained	26 ± 1.1	6 ± 2	14 ± 2	20 ± 3	22 ± 3	12 ± 3	18 ± 1
Theoretical				16.2	20.1	9.9	15.4

## Data Availability

Not applicable.

## Supplementary Materials

The following supporting information can be downloaded at: https://www.mdpi.com/article/10.3390/pharmaceutics14020240/s1, Figure S1: Examples of lipids that were able to solubilize the NC (i), while the (ii) figures are representatives of the ones that could not solubilize the NC, since compound crystals can be visualized, Figure S2: Images of the lipids that solubilized the NC, after melting and after cooling down, Figure S3: Images of the samples before and after the first and second centrifugation included in the accelerated stability study assays before centrifugation (i); after the first centrifugation (30 min) (ii); and after the second centrifugation (30 min + 30 min) (iii), Table S1: Solid Lipid Screening, Table S2: Ultra-Turrax and ultrasonication times tested for Gelucire^®^ 50/13 and Pluronic F-127, and the formulation characteristics (size, zeta potential (ZP) and PdI), Table S3: Ultra-Turrax and ultrasonication times tested for Gelucire^®^ 50/13 and Tween 80, and the formulation characteristics (size, zeta potential (ZP) and PdI), Table S4: Ultra-Turrax and ultrasonication times tested for Compritol^®^ HD5 ATO and Pluronic F-127, and the formulation characteristics (size, zeta potential (ZP) and PdI), Table S5: Ultra-Turrax and ultrasonication times tested for Compritol^®^ HD5 ATO and Tween 80, and the formulation characteristics (size, zeta potential (ZP) and PdI).
