# Supplementary material for: Vine Cane Compounds to Prevent Skin Cells Aging through Solid Lipid Nanoparticles
Source: Pharmaceutics. 2022 Jan 20;14(2):240. doi: 10.3390/pharmaceutics14020240 (PMC8876727; doi:10.3390/pharmaceutics14020240)
Supplement: Supplementary file 1 [file pharmaceutics-14-00240-s001.zip › pharmaceutics-1526703-supplementary.pdf]

Supplementary Material: Vine Cane Compounds to Prevent Skin Cells Aging through Solid Lipid Nanoparticles

Adriana Pereira, Maria João Ramalho, Renata Silva, Vera Silva, Rita Marques-Oliveira, Ana Catarina Silva, Maria Carmo Pereira and Joana A. Loureiro

Table S1. Solid Lipid Screening

	**EGCG**					**Resveratrol**					**Myricetin**				
		**Time (min)**					**Time (min)**					**Time (min)**			
**Solid lipid**	**Ratio (w/w)**	**15**	**30**	**45**	**60**	**Ratio (w/w)**	**15**	**30**	**45**	**60**	**Ratio (w/w)**	**15**	**30**	**45**	**60**
**Gelucire® 50/13**	1:100	-	-	-	-	1:100	-	-	-	-	1:100	-	-	-	-
**Compritol® H105 ATO**	1:100	-	-	-	-	1:200	+	+	+	-	1:100	-	-	-	-
**Softisan® 100**	1:100	-	-	-	-	1:200	+	+	+	+					
**Apifil®**	1:100	+	+	+	+										
**Precirol® 5 ATO**	1:100	+	+	+	+										
**Witepsol® E76**	1:100	+	+	+	+										

**Suppocire DM Pellets**	1:100	+	+	+	+		
**Suppocire NA15 pellets**	1:100	+	+	+	+		
**Gelucire 39/01**	1:100	+	+	+	+		
**Gelucire® 43/01**	1:100	+	+	+	+		
**Cetyl palmitate**	1:100	+	+	+	+		
**Stearic acid**	1:100	+	+	+	+		
**Dynasan® 118**	1:100	+	+	+	+		
**Glyceryl monostearate**	1:100	+	+	+	+		
**Softisan® 154**	1:100	+	+	+	+		

Table S2. Ultra-Turrax and ultrasonication times tested for Gelucire® 50/13 and Pluronic F-127, and the formulation characteristics (size, zeta potential (ZP) and PdI).

SLN: Gelucire® 50/13 + pluronic-F127		
S (min) \ UT(min)	0,5	2
5	Consistency and colour not acceptable	-
15	Consistency and colour not acceptable	-
30	Consistency and colour not acceptable	-

Table S3. Ultra-Turrax and ultrasonication times tested for Gelucire® 50/13 and Tween 80, and the formulation characteristics (size, zeta potential (ZP) and PdI).

SLN: Gelucire® 50/13 + Tween 80		
S (min) \ UT(min)	0,5	2
5	Consistency and colour not acceptable	-
15	-	-
30	Consistency and colour not acceptable	Consistency and colour not acceptable

Table S4. Ultra-Turrax and ultrasonication times tested for Compritol® HD5 ATO and Pluronic F-127, and the formulation characteristics (size, zeta potential (ZP) and PdI).

SLN: Compritol® HD5 ATO + pluronic-F127		
S (min) \ UT(min)	0,5	2
5	**Size:** 83%- 716 nm and 16%- 138 nm<br>**PdI:** 0,409<br>**ZP:** -0,6 mV	**Size:** 350 ± 17 nm<br>**PdI:** 0,353 ± 0,007<br>**ZP:** -0,4 ± 0,3 mV
15	-	**Size:** 206 ± 5 nm<br>**PdI:** 0,21 ± 0,01<br>**ZP:** -1,9 ± 0,4 mV
30	**Size:** 315 ± 10 nm<br>**PdI:** 0,434 ± 0,003<br>**ZP:** -0,5 ± 0,1 mV	**Size:** 96 ± 33 nm<br>**PdI:** 0,307 ± 0,004<br>**ZP:** -1,7 ± 0,7 mV

Table S5. Ultra-Turrax and ultrasonication times tested for Compritol® HD5 ATO and Tween 80, and the formulation characteristics (size, zeta potential (ZP) and PdI).

SLN: Compritol® HD5 ATO + tween 80

S (min) \ UT(min)	0,5	2
5	**Size:** 81,5%- 1844 nm and 18,5%- 90 nm<br>**PdI:** 0,631<br>**ZP:** 37,1%-(-13,1) mV and 62,9%-14,8 mV	-
15	-	Solidified
30	**Size:** 92,2%- 2025 nm and 7,8%- 63 nm<br>**PdI:** 0,560<br>**ZP:** -6,39 mV	Solidified

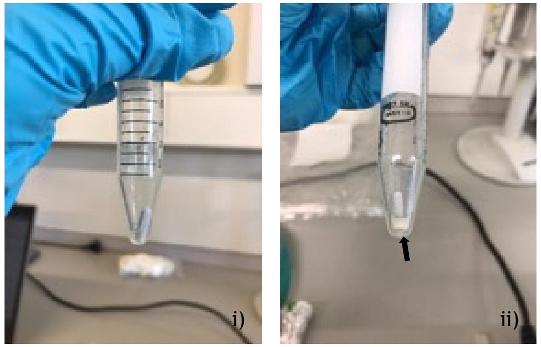

Solid lipid screening for EGCG in a 1:100 ratio: i) compritol HD5 ATO and ii) Glyceryl monostearate

Solid lipid screening for Resveratrol in a 1:200 ratio: i) compritol HD5 ATO and ii) softisan 100	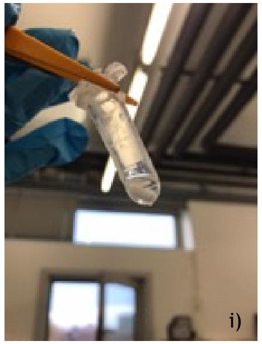 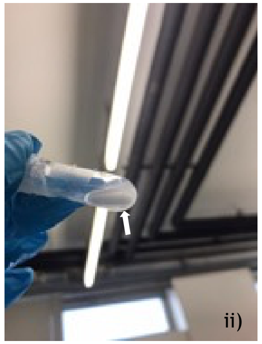
Solid lipid screening for myricetin in a 1:100 ratio: i) compritol HD5 ATO. All the tested solid lipids were compatible with this natural compound	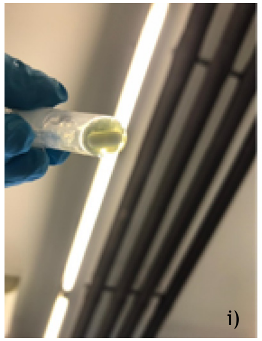

Figure S1. Examples of lipids that were able to solubilize the NC (i), while the (ii) figures are representatives of the ones that could not solubilize the NC, since compound crystals can be visualized.

Lipids / Conditions	NC	Melted	After cooling down
Gelucire 50/13; 15 min; ratio 1:100		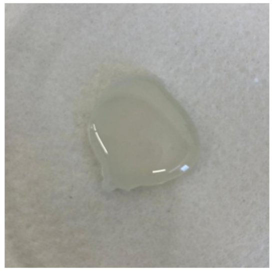	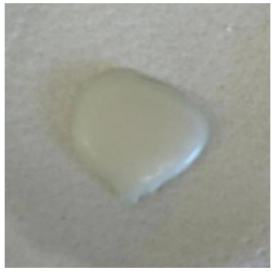
Compritol HD5 ATO; 15 min; ratio 1:100	EGCG	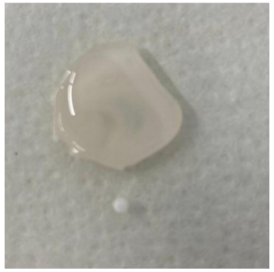	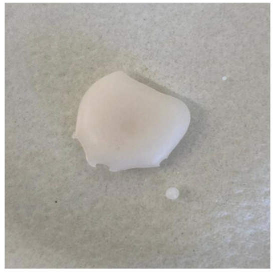
Softisan 100; 15 min; ratio 1:100		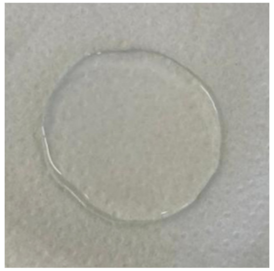	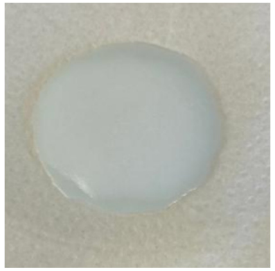

Gelucire 50/13; 15 min; ratio 1:100	Resveratrol	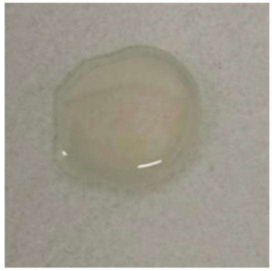	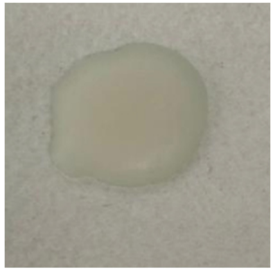
Compritol HD5 ATO; 60 min; ratio 1:200		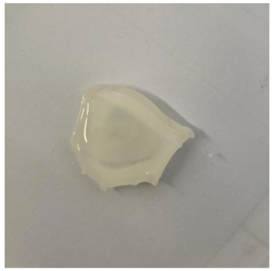	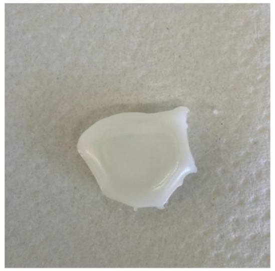
Gelucire 50/13; 15 min; ratio 1:100	Myricetin	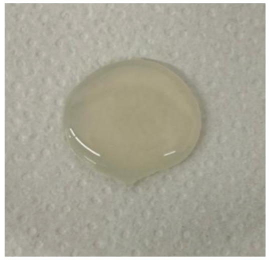	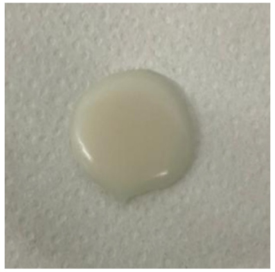
Compritol HD5 ATO; 15 min; ratio 1:100		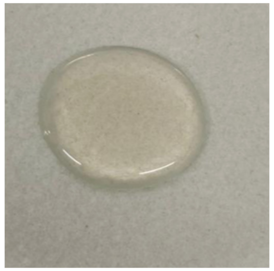	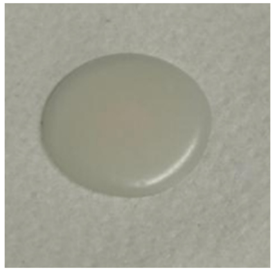

Figure S2. Images of the lipids that solubilized the NC, after melting and after cooling down.

Epigallocathechin gallate-loaded SLNs	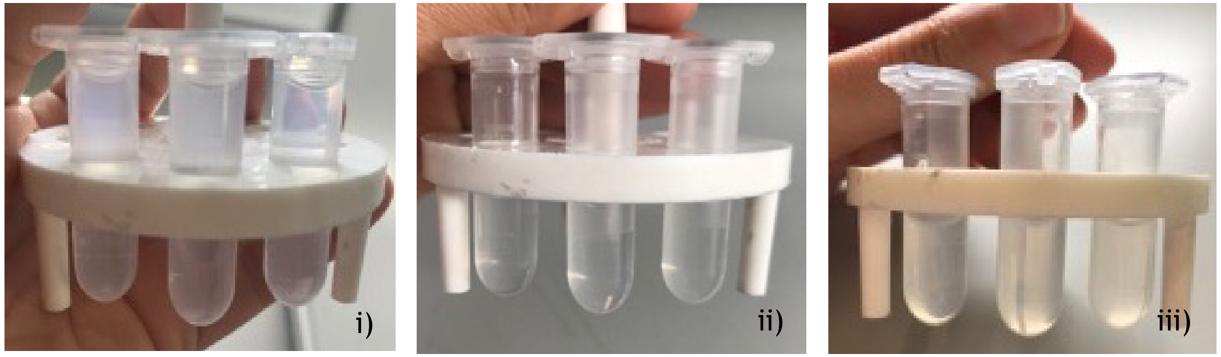
Resveratrol-loaded SLNs	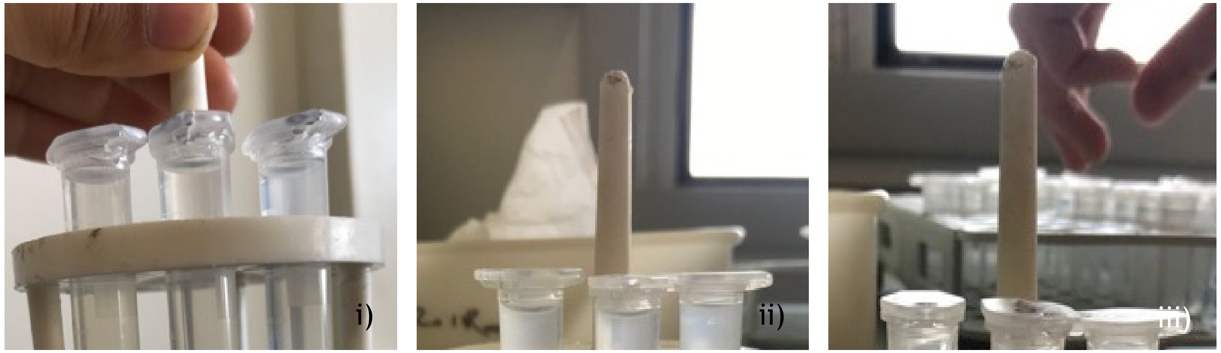

Figure S3. Images of the samples before and after the first and second centrifugation included in the accelerated stability study assays before centrifugation (i); after the first centrifugation (30 minutes) (ii); and after the second centrifugation (30 minutes + 30 minutes) (iii).
